# Robotically assisted enhanced-view totally extraperitoneal repair (eTEP) of a recurrent umbilical hernia in a patient with peritoneal dialysis

**DOI:** 10.3205/iprs000158

**Published:** 2021-06-09

**Authors:** Katrin Bauer, Frank Heinzelmann, Robert Vogel, Peter Büchler, Björn Mück

**Affiliations:** 1 Abteilung für Allgemein-, Viszeral-, Thorax- und Kinderchirurgie, Klinikum Kempten, Klinikverbund Allgäu, Kempten, Germany

**Keywords:** robotic ventral hernia repair, eTEP, peritoneal dialysis, umbilical hernia, retromuscular mesh placement

## Abstract

**Background:** Abdominal wall hernias are frequent in patients with peritoneal dialysis. Guidelines recommend an open hernia repair with extraperitoneal mesh placement to avoid access to the abdominal cavity.

**Method:** We performed a lateral docking robotically assisted enhanced-view totally extraperitoneal repair (eTEP) of a recurrent umbilical hernia with diastasis recti in a patient with peritoneal dialysis due to polycystic kidney disease. After suturing of the midline a 20 x 28 cm mesh was placed in the retrorectus space, covering the whole area of preparation while also overlapping all trocar sites. A drainage was left in the retrorectus space until the first session of PD did not sample any form of leakage.

**Result:** Robotically assisted totally extraperitoneal hernia repair was feasible. The patient was able to continue peritoneal dialysis without intermittent hemodialysis. There was no leakage of the dialysate to the retrorectus space. Postoperative recovery was uneventful. 6 months after surgery the patient was free from pain and showed no signs of recurrence.

**Conclusion:** Robotically assisted totally extraperitoneal hernia repair in patients with umbilical hernia and peritoneal dialysis could be a promising surgical technique to combine the advantages of minimally-invasive surgery with totally extraperitoneal mesh placement without access to the abdominal cavity.

## Zusammenfassung

**Hintergrund:** Die Entwicklung einer Umbilicalhernie ist eine häufige Komplikation bei Patienten mit Peritonealdialyse. In den Leitlinien wird eine offene Hernioplastik mit extraperitonealer Netzeinlage empfohlen, ohne die Bauchhöhle zu eröffnen.

**Methoden:** Wir versorgten die Rezidivnabelhernie eines Patienten mit Peritonealdialyse aufgrund einer polyzystischen Nierenerkrankung in robotisch-assistierter total extraperitonealer Technik mit lateralem Zugang. Nach Nahtrekonstruktion der Mittellinie wurde ein 20 x 28 cm großes Netz in den Retrorektalraum eingebracht, welches das gesamte Operationsgebiet inklusive aller Trokarzugänge abdeckte. Eine Drainage wurde im Netzlager belassen, bis die erste Peritonealdialyse keine Undichtigkeit der Peritonealhöhle zeigte.

**Ergebnis:** Die Operation konnte erfolgreich in robotisch-assistierter, total extraperitonealer Technik durchgeführt werden. Die Peritonealdialyse konnte ohne intermittierende Hämodialyse fortgesetzt werden. Es zeigte sich kein Austritt von Dialysat in das Netzlager. Der perioperative Verlauf war komplikationslos. Sechs Monate nach dem Eingriff war der Patient beschwerde- und rezidivfrei.

**Schlussfolgerung:** Die robotisch-assistierte total extraperitoneale Hernienoperation von Patienten mit Umbilicalhernie und Peritonealdialyse ist ein vielversprechendes Verfahren, welches die extraperitoneale Netzplatzierung mit den Vorteilen eines minimal-invasiven Zugangs vereint.

## Introduction

Peritoneal dialysis (PD) is frequently associated with abdominal wall hernias. Last year European Hernia Society (EHS) and American Hernia Society (AHS) published guidelines, focusing on primary ventral hernia repair in PD [1]. Open repair using onlay or preperitoneal mesh placement without access to the peritoneal cavity was recommended. Recently new extraperitoneal techniques with minimal invasive mesh placement in the retrorectal space were established in hernia surgery.

## Case description

**Figure 1:**
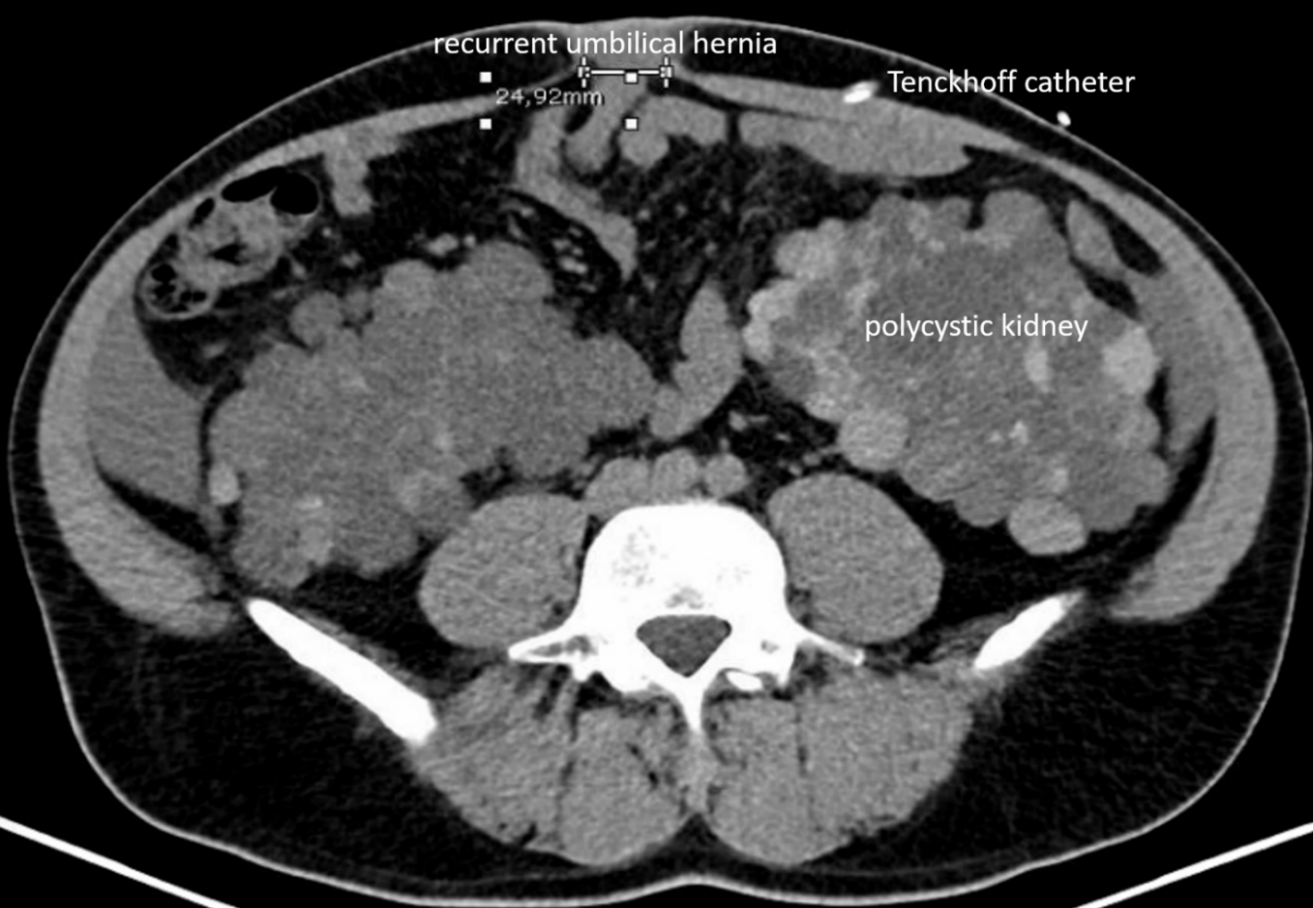
CT scan preoperative

**Figure 2:**
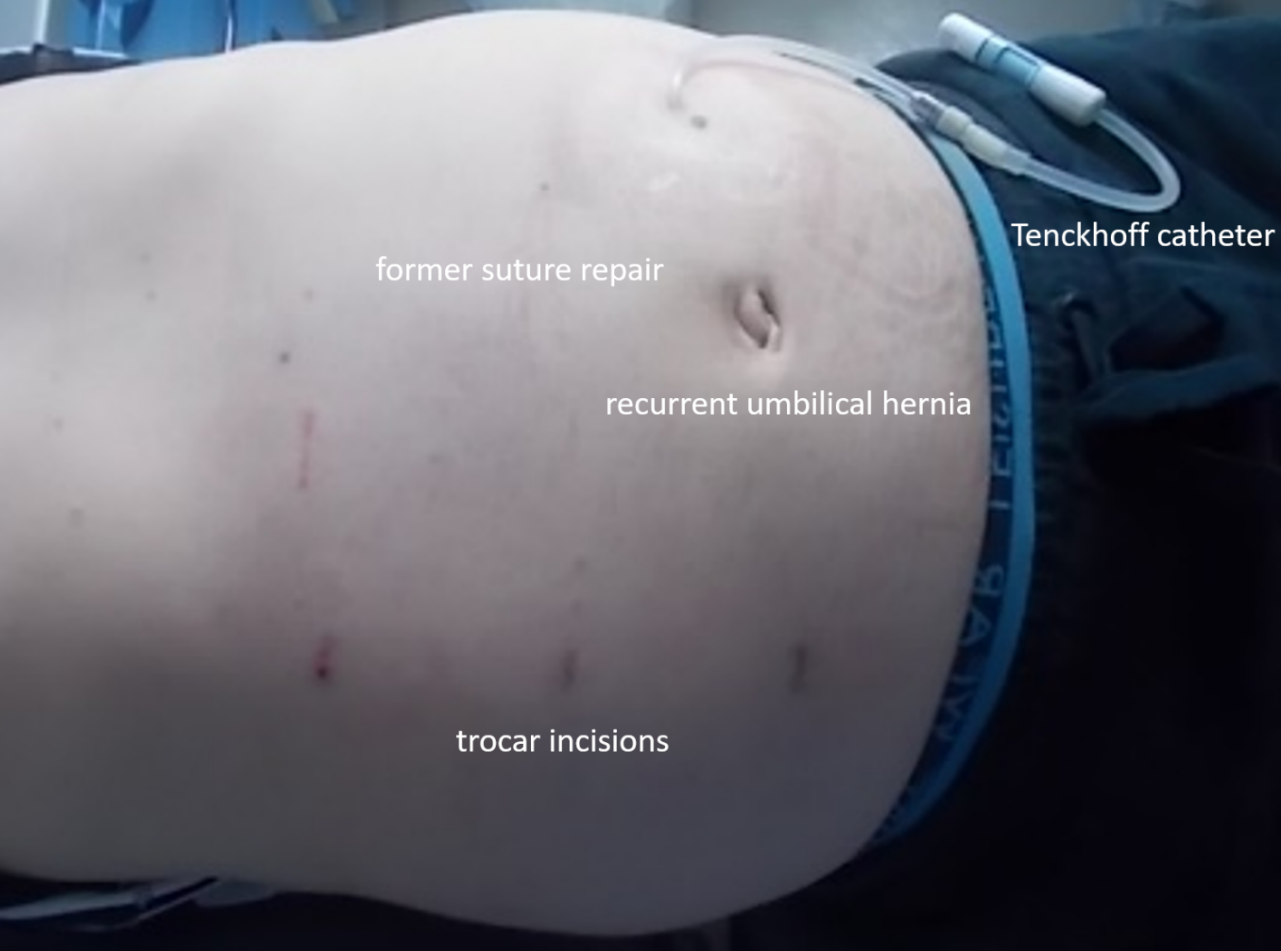
Postoperative picture of the abdominal wall

We report a case of a robotically assisted enhanced-view totally extraperitoneal hernia repair (eTEP) of a recurrent umbilical hernia, performed on a 43-year-old male patient with polycystic renal disease and PD. The patient presented with a 2.5 x 2 cm recurrent umbilical hernia, associated with a 3.5 cm diastasis recti and an additional epigastric hernia. The year before suture repair of aforementioned umbilical hernia was performed during the implantation of the Tenckhoff catheter. An additional suture repair of another epigastric hernia between both hernia localisations prior to implantation of the catheter was reported. The patient had a BMI of 31 and no further comorbidities other than epilepsy. Ultrasound examinations showed that the catheter was implanted close to the umbilicus while passing the retrorectal space more caudally (Figure 1 (Fig. 1), Figure 2 (Fig. 2)). Hence we concluded that a retrorectal mesh placement with an overlap of at least 5 cm should be possible. 

Regarding the necessity of regular peritoneal dialysis, we established following problems:

- Due to peritoneal dialysis abdominal pressure is elevated, resulting in a higher risk of hernia recurrence.
- The anticipated area of mesh placement is compromised by the catheter crossing the abdominal wall while also increasing the risk of infection.
- Intraperitoneal mesh placement is not favoured in patients undergoing peritoneal dialysis.
- The peritoneal cavity is altered by PD in a way that makes it less accessible to surgery and is also accompanied by an increase in morbidity.
- An appropriate mesh covering the whole epigastrium including the diastasis recti, the concomitant epigastric hernia as well as the trocar sites is obligatory.

In coherence with all the aforementioned, we decided that an eTEP hernia repair, with an uncoated mesh placed in the retrorectal space, would be a suitable procedure. 

**Figure 3:**
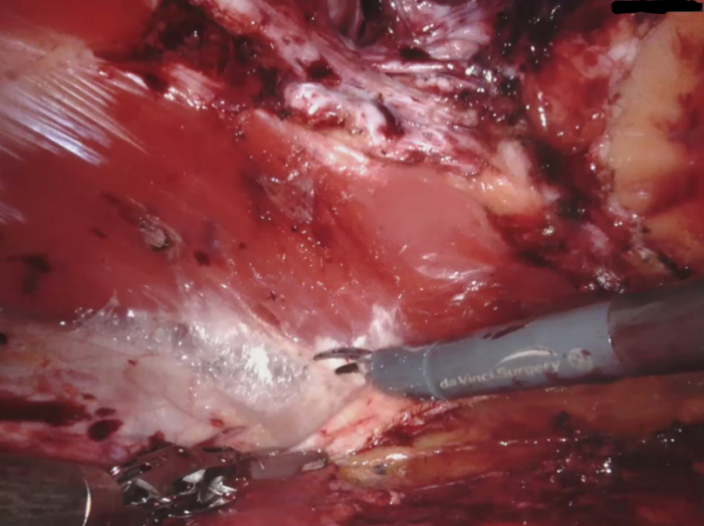
Intraoperative view of the dissected left rectus sheath

**Figure 4:**
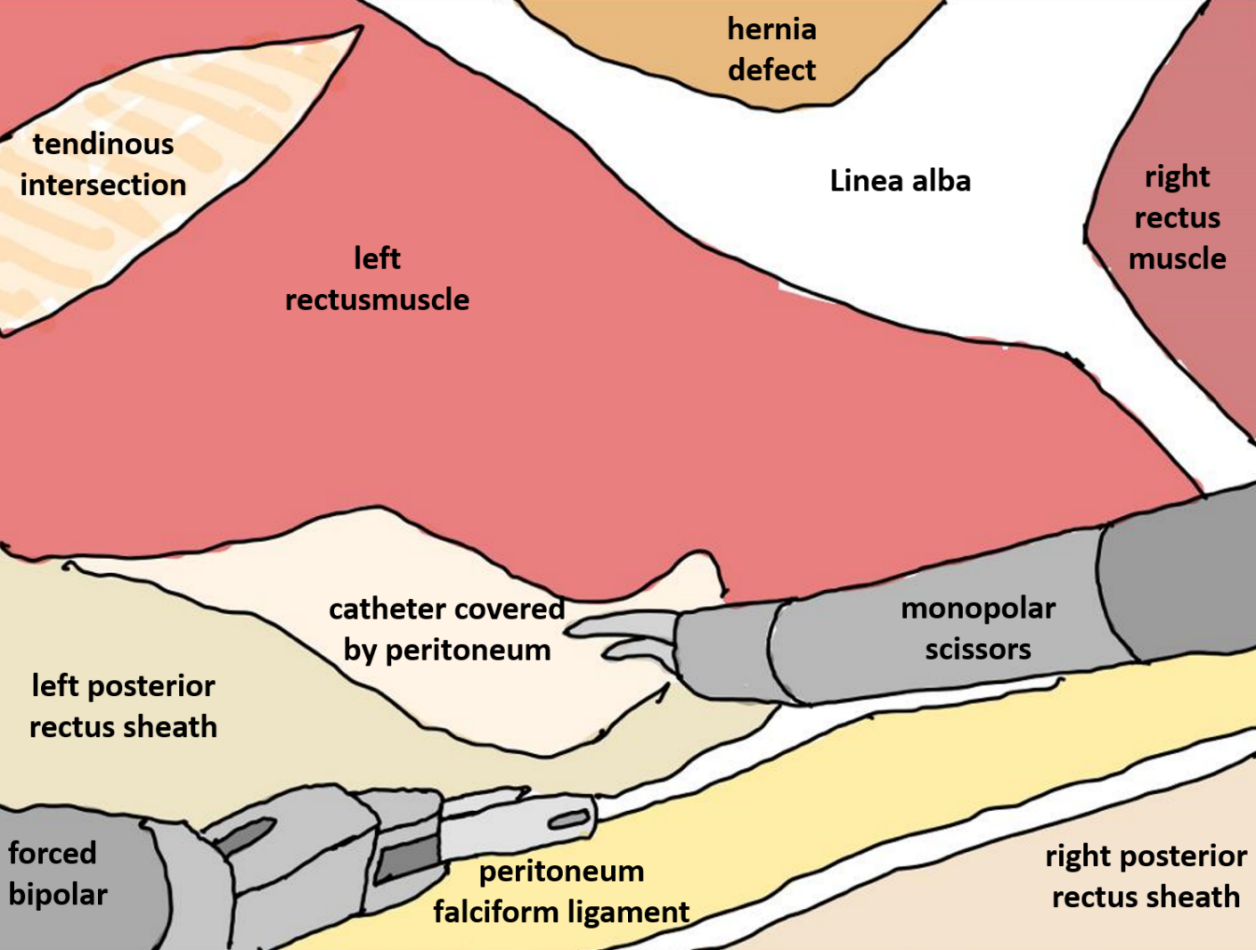
Illustration with anatomic landmarks

The right retrorectus space was entered with an optic trocar. After blunt dissection with the camera the first DaVinci trocar was placed medially to the right semilunar line. During endoscopic dissection two more trocars were placed in the same line and the DaVinci X system was docked coming from the left side of the patient. Crossover to the left retrorectal space was started with an incision of the right posterior rectus sheath laterally to the linea alba. The preperitoneal fat of the ligamentum falciforme was separated from the linea alba and the left rectus sheath was opened by an incision of the left posterior rectus sheath. During crossover the hernias were repositioned and a small opening of the peritoneum was sutured. Dissection was proceeded in a lateral direction towards the left semilunar line until the catheter covered by a membrane of peritoneum could be visualised (Figure 3 (Fig. 3), Figure 4 (Fig. 4)). After suturing the diastasis recti and the two hernias with a resorbable barbed suture, a 20 x 28 cm PVDF mesh was placed in the connected retrorectal spaces, covering the whole area of preparation while also overlapping all trocar sites. The operation was completed placing a drain on top of the mesh and suturing of the skin. 

Postoperatively PD was paused for 72 h. The drain remained in place until the first session of PD did not sample any form of dialysate in the drain. Over 6 weeks a reduced amount of dialysate was used. Clinically and sonographically there were no signs of recurrence 6 months after surgery whilst PD was able to be performed with its preoperative parameters.

## Discussion

In 2020 the EHS and AHS focused on the treatment of umbilical hernias occurring before or during PD in their guidelines for treatment of primary ventral hernias in rare locations or special circumstances [1]. Various retrospective case series have reported an occurrence rate of 3 to 15 percent of umbilical hernias in patients undergoing PD [2], [3], [4]. Umbilical hernia was the most common defect, followed by inguinal and epigastric hernias [2], [5]. The development of a hernia represents a frequent complication in PD, claiming up to 60.4% of all anatomical complications [6]. Risk factors are discussed controversially. However, the ones most frequently mentioned include: male gender, older age, multiparity, low body mass index, polycystic renal disease and prolonged PD duration [3], [4], [6], [7]. 

A few case series were published stating that simultaneous ventral hernia repair and peritoneal catheter placement seems to be a reliable and safe surgical procedure [2], [8]. The recently published guidelines are recommending the repair of a preexisting umbilical hernia before initiating PD [1]. However, strength of recommendation is weak and quality of evidence is low. So far there are neither RCTs nor any review articles on the treatment of hernias in PD patients.

Thomas et al. [9] examined if watchful waiting is an appropriate option for PD patients with asymptomatic ventral hernias. Most of the hernias in this single center study were localized at the umbilicus (78%). The cumulative incidence of ventral hernia repair was 13% and 21% within 12 and 24 months after PD catheter application. The authors concluded that watchful waiting may be an acceptable option for selected patients with asymptomatic ventral hernias at the time of initial PD catheter placement.

Articles about management of ventral hernias occurring during PD are rare. There are one review and a few case series recommending tension free mesh repair to continue PD [3], [5], [10], [11]. Usually intermittent hemodialysis is not necessary [5]. AHS and EHS guidelines recommend an open extraperitoneal repair with placement of a preperitoneal or onlay mesh without access to the peritoneal cavity, in order to avoid port-site hernias, fluid leakage from port sites and intraperitoneal mesh placement [1]. This technique is safe and associated with low morbidity. Studies evaluating the role of laparoscopic hernia repair don’t exist. Mesh augmentation is recommended due to the enlarged intra-abdominal pressure during PD. Martinez-Mier report a recurrence rate of 12% without implanting a mesh, compared with 0% in patients with mesh hernioplasty of 58 hernias in 50 patients under PD [11].

A novel approach using the eTEP technique for endoscopic retromuscular hernia repair [12] was evaluated by Belyansky et al. in 2017. A robotic modification of this technique was published in 2018 [13]. A robotically assisted eTEP hernia repair with retrorectal mesh placement in hernia patients undergoing PD has not been published so far. This approach could combine the advantages of minimally invasive surgery without access to the peritoneal cavity with an extraperitoneal mesh overlapping all trocar sites. The excellent visibility and the possibility of accurate preparation offered by the robotic system could contribute to the continuation of PD and hence avoiding unnecessary hemodialysis. Due to the high incidence of umbilical hernias in patients undergoing PD, the insertion of the dialysis catheter should be performed with sufficient distance to the umbilicus in order to enable an overlapping mesh placement.

## Conclusion

Robotic total extraperitoneal hernia repair in patients with umbilical hernia under peritoneal dialysis could be a promising surgical technique to combine the advantages of minimal-invasive surgery with totally extraperitoneal mesh placement without access to the abdominal cavity.

## Notes

### Competing interests

The authors declare that they have no competing interests.
